# High-dose exposure to butylparaben impairs thyroid ultrastructure and function in rats

**DOI:** 10.1038/s41598-024-55096-4

**Published:** 2024-02-24

**Authors:** Qi-Lan Jiang, Sha Li, Yang Zeng, Bo-Tao Zhang, Yu Cao, Tao Li, Jun Jiang

**Affiliations:** 1 Department of General Surgery (Thyroid Surgery), The Affiliated Hospital of Southwest Medical University, Luzhou, 646000 Sichuan Province China; 2 Department of Clinical Nutrition, The Affiliated Hospital of Southwest Medical University, Luzhou, 646000 Sichuan Province China; 3 Department of Orthodontics, The Affiliated Stomatology Hospital of Southwest Medical University, Luzhou, 646000 Sichuan Province China; 4 Key Laboratory of Medical Electrophysiology of Ministry of Education, Collaborative Innovation Center for Prevention and Treatment of Cardiovascular Disease, Institute of Cardiovascular Research, Southwest Medical University, Luzhou, 646000 Sichuan Province China

**Keywords:** Parabens (PBs), Butylparaben (BuP), Thyroid, Hypothyroidism, Rat, Endocrinology, Environmental impact

## Abstract

Parabens (PBs) are a class of preservatives commonly used in cosmetics and pharmaceuticals. Studies have shown that these compounds may act as endocrine disruptors, affecting thyroxine levels in humans. PBs with longer chain substituents, such as butylparaben (BuP), are less prone to complete biotransformation and are therefore more likely to accumulate in the body. In this study, the effect of high-dose exposure to BuP on thyroid microstructure, ultrastructure, and function was investigated in rats. 50 mg/kg bw per day of BuP was injected subcutaneously into the neck of rats for 4 weeks. Rat thyroid weight, microstructure, and ultrastructure were determined, and the levels of thyroid sodium/iodide symporter (NIS), serum thyroid hormones, and thyroid autoantibodies were measured. The human thyroid cell line was used to study the mechanism of BuP on thyroid epithelial cells. The weight of the thyroid gland of BuP-exposed rats was increased, the structure of the thyroid follicles was irregular and damaged, the mitochondria and rough endoplasmic reticulum were swollen and damaged, and the microvilli at the tip of the epithelium were reduced and disappeared. Serum total T3, total T4, free T3, and free T4 were decreased in BuP-exposed rats, and TSH, peroxidase antibody, and thyroglobulin antibody were increased. In vitro, BuP decreased the level of NIS in thyroid epithelial cells, inhibited proliferation and viability, and induced apoptosis in a dose-dependent manner. This study demonstrated that high-dose exposure to BuP induced structural, ultrastructural, and functional impairment to the thyroid gland of rats, which may be one of the factors leading to hypothyroidism.

## Introduction

Parabens (PBs) are a class of preservatives and antibacterial agents widely used in cosmetics, personal care products, pharmaceuticals and foods^1–3^. They have the advantages of broad-spectrum antibacterial activity, low allergenicity, high stability, no volatility and low production cost. According to the different ester groups, PBs can be divided into methylparaben (MeP), ethylparaben (EtP), propylparaben (PrP), butylparaben (BuP) and benzylparaben (BzP). Due to the widespread use, PBs are gradually becoming widely distributed in soil, air, surface water, sediment, and living organisms^4–7^. It is estimated that a person weighing 60 kg is exposed to approximately 76 mg of PBs (1.26 mg/kg) per day, of which 66% comes from personal care products and cosmetics, 33% from pharmaceuticals, and 1% from food^3^.

PBs enter the human body through the gastrointestinal tract, skin and respiratory tract. Most are hydrolyzed in the liver by carboxylesterase to ethanol and hydroxybenzoic acid (PHBA), which combines with glucuronic acid, glycine, and sulfate^8^. PBs absorbed through the skin are partially hydrolyzed in the skin^9^. Most PBs are excreted in urine, bile and feces, with 2% remaining in the body. As endocrine disrupting chemicals (EDCs), PBs are often classified as low accumulation compounds. Short-chain PBs are almost 100% completely metabolized in a short period of time, while long-chain parabens, such as BuP, take longer to metabolize and are not easily completely biotransformed^10–12^.

PBs possess estrogen-like activity and therefore show endocrine disrupting effects^13–15^ that are toxic to the mammary, adrenal and ovarian etc. al^16–19^. PBs also disrupt thyroid function. BuP disrupts the hypothalamic-pituitary-thyroid axis and reduces thyroid hormone levels at early developmental stages in zebrafish^20^. In mammals, PBs have been shown to increase thyroid volume in rats^21^. Population studies suggest that individual exposure to PBs may be associated with the risk of benign thyroid nodules and cancer^22^. PBs also interfere with thyroid hormone metabolism in humans^23,24^. However, the exact mechanism by which PBs induce changes in thyroid function is unclear.

In this study, the effect of high-dose exposure to BuP on the rat thyroid was investigated. The reason for choosing BuP is that it has been reported to affect the development of the hypothalamic-pituitary-thyroid axis in zebrafish^20^. In addition, as mentioned above, BuP is metabolized relatively slowly in the body due to its long-chain nature and is therefore more likely to have a cumulative effect^10–12^.

## Materials and methods

The animal experiments were ethically approved by the Laboratory Animal Center of Southwest Medical University. All procedures complied with the Guide and Care and Use of Laboratory Animals (U.S. National Institutes of Health, 1996) and the China Animal Management Regulations (Chinese Ministry of Health Document No. 55, 2001). The study is reported according to the ARRIVE guidelines.

### Animal model

**Figure 1:**
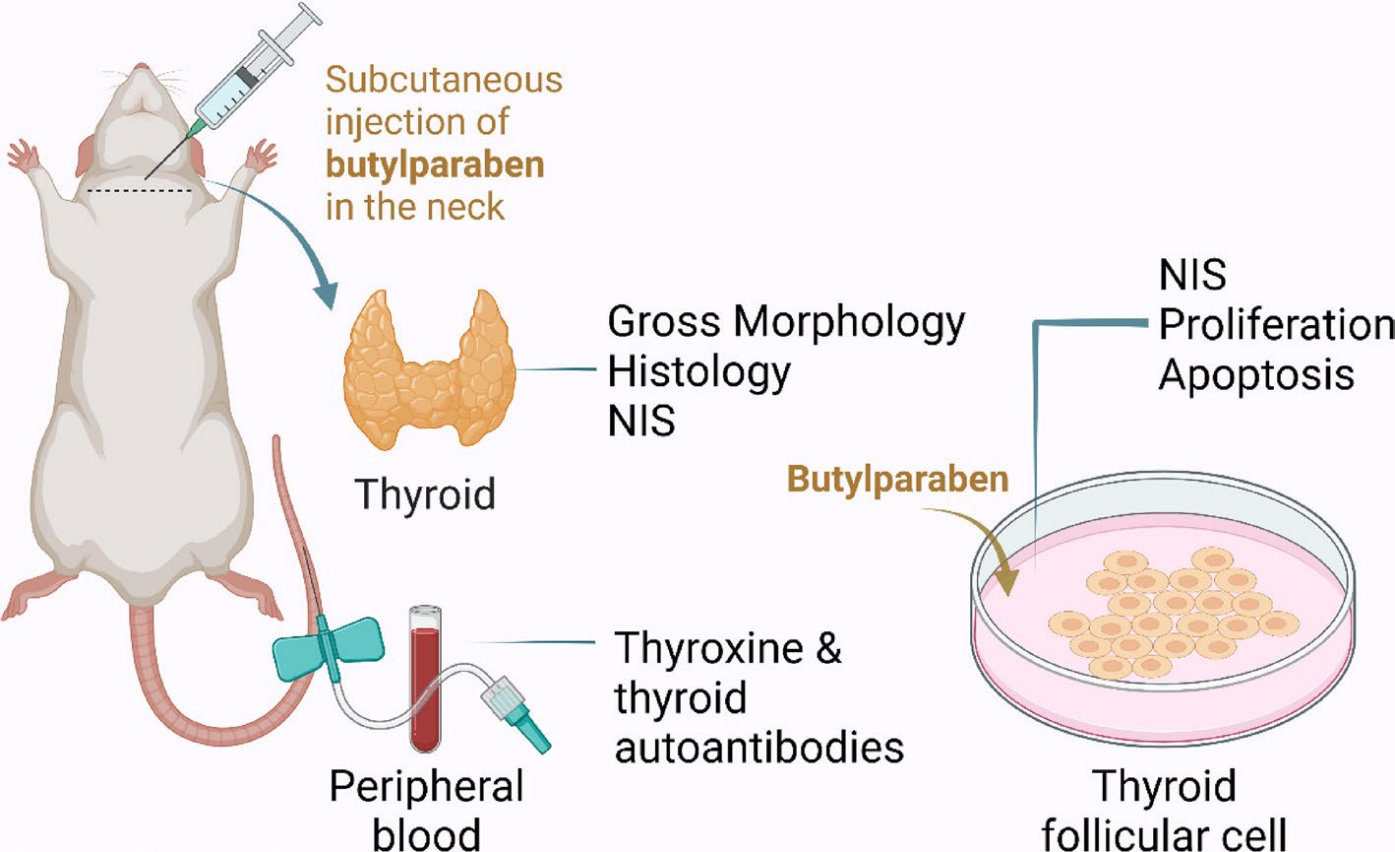
Experimental procedure. This study included animal and cell experiments. In vivo, rats were injected subcutaneously with BuP at 50 mg/kg bw/d for 4 weeks. After euthanasia, thyroid histology, serum levels of thyroid hormones, and thyroid autoantibodies were determined. In vitro, the human thyroid follicular cell line Nthy cells were exposed to 0 to 80 mg/L BuP and cell viability, proliferation, apoptosis, and sodium iodide symporter (NIS) levels were determined.

A total of 16 8-week-old healthy male Sprague–Dawley rats were randomly assigned to the control and the BuP exposure group (n = 8/each). First, 200 mg of butyl paraben (Solarbio, Beijing, China. Catalog No. IB0390) was dissolved in 30 ul of DMSO (Sigma-Aldrich, US. Catalog No. 67–68-5) and then diluted with ultrapure olive oil (PythonBio, Guangzhou, China. Catalog No. AAPR144) to a concentration of 200 mg/ml to obtain a BuP working fluid. In the BuP exposure group, rats were injected subcutaneously in the neck at a dose of 50 mg/kg body weight/day for 4 weeks. The concentration and duration of treatment were selected based on previous studies^21,25,26^. For mice, the LD50 (subcutaneous) of BuP is > 2500 mg/kg (11.56 mmol/kg). For Sprague–Dawley rats, the no observed adverse effect level (NOAEL) and no observable effect level (NOEL) of BuP are 100–1000 mg/kg bw/day, and the lowest observable effect level (LOEL) is 100 mg/kg bw/day (https://ntp.niehs.nih.gov/sites/default/files/ntp/htdocs/chem_background/exsumpdf/butylparaben_508.pdf). In the control group, rats were injected with the same volume of DMSO olive oil for the same duration. At the end of the animal experiment, the rats were euthanized according to the AVMA Guidelines for the Euthanasia of Animals (2020 Edition). Rats were anesthetized in a chamber containing 5% isoflurane oxygen and euthanized by cervical dislocation. Peripheral blood serum and thyroid glands were collected (Fig. 1).

### Gross observation and histologic examination of rat thyroid gland

The rat thyroid was carefully dissected from the trachea and weighed. At the same time, the body weight of the rats was recorded and the ratio of thyroid weight to body weight was calculated. Thyroid glands were fixed in 4% paraformaldehyde for 24 h, embedded in paraffin, and sectioned at 4 μm. The sections were subjected to hematoxylin–eosin staining, and the microstructure of thyroid follicles was observed by light microscopy (Nikon Eclipse Ci-L, Tokyo, Japan).

### Ultrastructural observation of rat thyroid follicular cells

Fresh thyroid glands were fixed in 2.5% glutaraldehyde for 24 h at 4 °C and 1% osmic acid for 2 h. The glands were embedded in resin and cut into 60- to 80-nm ultrathin sections (Leica EM UC7 ultramicrotome, Wetzlar, Germany). The sections were stained with 2% uranyl acetate and 2.6% lead citrate solution, and the cell ultrastructure was observed by transmission electron microscopy (Hitachi HT7700, Tokyo, Japan).

### Determination of thyroid hormones and thyroid autoantibodies in rat serum

The levels of total triiodothyronine (TT3), total thyroxine (TT4), free T3 (FT3), free T4 (FT4), thyroid-stimulating hormone (TSH), thyroid peroxidase antibody (TPO-Ab), and thyroglobulin antibody (Tg-Ab) were determined by enzyme-linked immunosorbent assay (ELISA). The TT3 ELISA kit, catalog no. JL13028, was purchased from Shanghai Jianglai Biological Technology. The FT3 ELISA kit, catalog no. FSEA2126, was purchased from Shanghai Fushen Biological Technology. The TT4 ELISA kit, FT4 ELISA kit, high sensitivity TSH (U-TSH) ELISA kit, TPO-Ab ELISA kit, and Tg-Ab ELISA kit, catalog No. ml059551, ml002849, ml059371, ml003369, and ml003044, were purchased from Shanghai Enzyme-linked Biotechnology. Thyroid hormones and autoimmune antibodies concentrations were determined according to the reagent manufacturer's instructions.

### In vitro experiments

Human thyroid follicular cell line Nthy-ori 3-1 cells (Catalog No. CC-Y1708, EK-BioScience, Shanghai, China.) were cultured in thyroid cell complete medium (Catalog No. CC-Y1708M, BioScience, Shanghai, China.). Thyroid follicular cells were identified by thyroglobulin immunofluorescence staining (thyroglobulin antibody: Beyotime, Wuhan, China. Catalog No. AG3385).

Cell passages 3 to 10 were used in the experiment. For BuP stimulation experiments, the Nyth cells were first treated with BuP at concentrations of 0 mg/L, 10 mg/L, 20 mg/L, 40 mg/L, 60 mg/L, and 80 mg/L for 24 h, and then subjected to the following assays.

### Cell proliferation and viability assays

The 5-ethynyl-2′-deoxyuridine (EdU) cell proliferation kit (RiboBio, Guangzhou, China. Catalog No. C10310) was used to determine cell proliferation. According to the manufacturer's instructions, EdU was infiltrated into the proliferating cells, and the ratio of proliferating cells was calculated by fluorescence microscopy.

Cell proliferation was also determined by Western blot analysis of proliferating cell nuclear antigen (PCNA). PCNA antibody (Cell Signaling Technology, MA, USA. Catalog No. 13110) and GAPDH antibody (Cell Signaling Technology. Catalog No. 2118) were used in the experiment. GAPDH was used as an internal control.

Cell viability was determined using the CCK8 detection kit (Beyotime, Wuhan, China. Catalog No. C0037) according to the reagent manufacturer's instructions. A microplate reader (iMark™ Microplate Absorbance Reader, Bio-Rad, CA, USA) was used to detect the absorbance of the sample at 450 nm (optical density at 450, OD 450). Cell viability was expressed as a percentage.

### Cell apoptosis assays

Apoptosis was determined by Western blotting to detect the relative levels of cleaved caspase-3 (Absin, Shanghai, China. Catalog No. ab131825), and the level of total caspase-3 was used as an internal control. The ratio of cleaved caspase-3 to total caspase-3 was used to evaluate cell apoptosis.

Apoptosis was also determined by Western blotting to detect the relative levels of Bcl-2 (ProeinTech, Chicago, USA. Catalog No. 12789-1-AP) and Bax (ProeinTech, Chicago, USA. Catalog No. 50599-2-Ig). GAPDH was used as an internal reference (GAPDH antibody purchased from Cell Signaling Technology. Catalog No. 2118). The ratio of Bcl-2 to Bax (Bcl-2/Bax) was used to evaluate cell apoptosis.

### Western blotting

The sodium iodide symporter (NIS) is a cell membrane protein located in the basolateral membrane of thyroid follicular cells and mediates iodine transport in the cells. The relative level of NIS was detected by Western blotting using its antibody (abcam, Cambridge, UK. Catalog No. ab199410). GAPDH (Cell Signaling Technology. Catalog No. 2118) was used as an internal reference.

Rat thyroid or human Nthy cells were pretreated with RIPA lysis buffer and protease inhibitors (Beyotime Biotechnology. Catalog No. P0013 and P1005) to extract total proteins. Total proteins were determined using a multimode microplate reader (type BioTek Synergy H1, Agilent, Inc.) and separated by 10% SDS-PAGE gel electrophoresis and transferred to PVDF membranes (Merck Millipore, MA, USA. Catalog No. IPVH00010). The PVDF membranes were blocked with BSA (Sigma-Aldrich. Catalog No. 9048-46-8) and then hybridized with antibodies. Finally, the PVDF membranes were incubated with a developing solution and developed in the Molecular Imager Gel Doc XR system (Bio-Rad Laboratories, Inc.). Western blots were analyzed with ImageJ software (National Institutes of Health).

### Statistical analysis

Each experiment was repeated at least three times (n ≥ 3). Experimental results were expressed as the mean ± standard deviation (SD). GraphPad Prism 9 (GraphPad, Inc., San Diego, CA, USA) was used for data analysis and to generate statistical graphs. A two-tailed Student's t-test was used for comparisons between the two groups. One-way analysis of variance (ANOVA) was used for comparisons between multiple groups. Fishers' least significant difference was used as a post hoc test. *P* < 0.05 was considered statistically significant.

## Results

### BuP caused enlargement of rat thyroid gland and follicular injury

**Figure 2:**
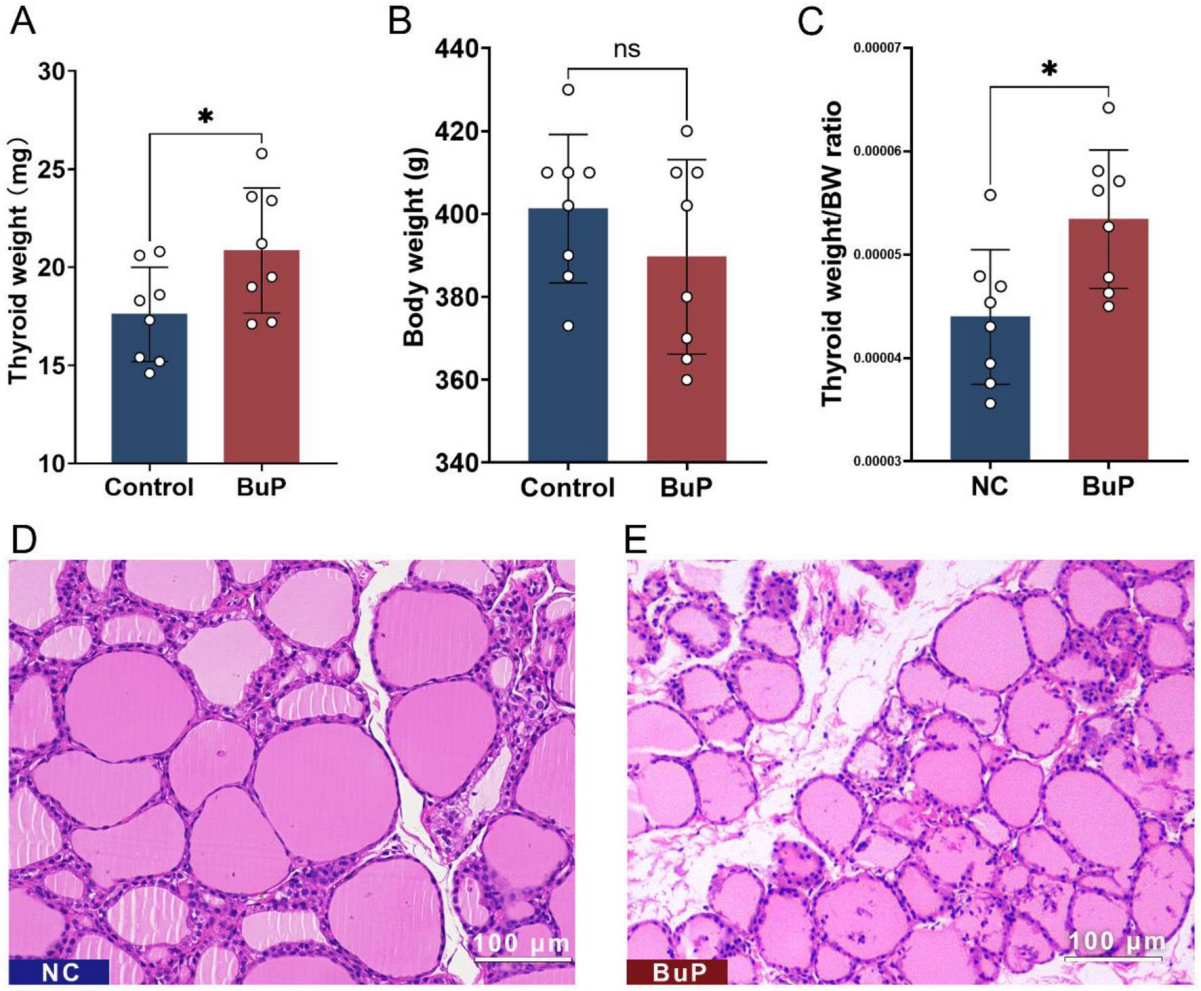
BuP caused thyroid enlargement and follicular damage in rats. (**A**) At the end of the animal experiment, the rats were euthanized and the thyroid glands were carefully dissected and weighed. The weight of the thyroid gland of the BuP-exposed rats was heavier than that of the control rats. (**B**) There was no significant difference in body weight between control rats and BuP-exposed rats. (**C**) The ratio of thyroid gland to body weight of BuP-exposed rats was higher than that of control rats. (**D**) In the control group, the normal thyroid follicles are uniform in size, the follicular wall is intact, and there is no inflammatory cell infiltration in the follicular stroma. (**E**) Thyroid follicles of BuP-exposed rats were inconsistent in size, follicle walls were incomplete, and there were many inflammatory cells infiltrating the follicular cavity and follicular stroma. n = 8, data are expressed as mean ± SD. Compared with control group, * *P* < 0.05. ns, not statistically significant. scale bar = 100 µm.

Dissection of the rat thyroid gland showed that the volume and weight of the thyroid gland increased in the BuP-exposed group (Fig. 2A). The body weights of rats in the control and experimental groups have no significant difference (Fig. 2B), therefore the ratio of thyroid weight to body weight was increased in the BuP-exposed group (Fig. 2C).

The microstructure of the rat thyroid was observed under the microscope. Compared with the thyroid follicles of the control group (Fig. 2D), the thyroid follicles in the BuP-exposed group were morphologically irregular, some thyroid epithelial cells were shed from the follicles, colloid in the follicles was reduced or absent, the local epithelium showed columnar hyperplasia, and many inflammatory cells infiltrate the thyroid follicular stroma and the follicular cavity (Fig. 2E).

### BuP induced ultrastructural injury of the thyroid epithelium

**Figure 3:**
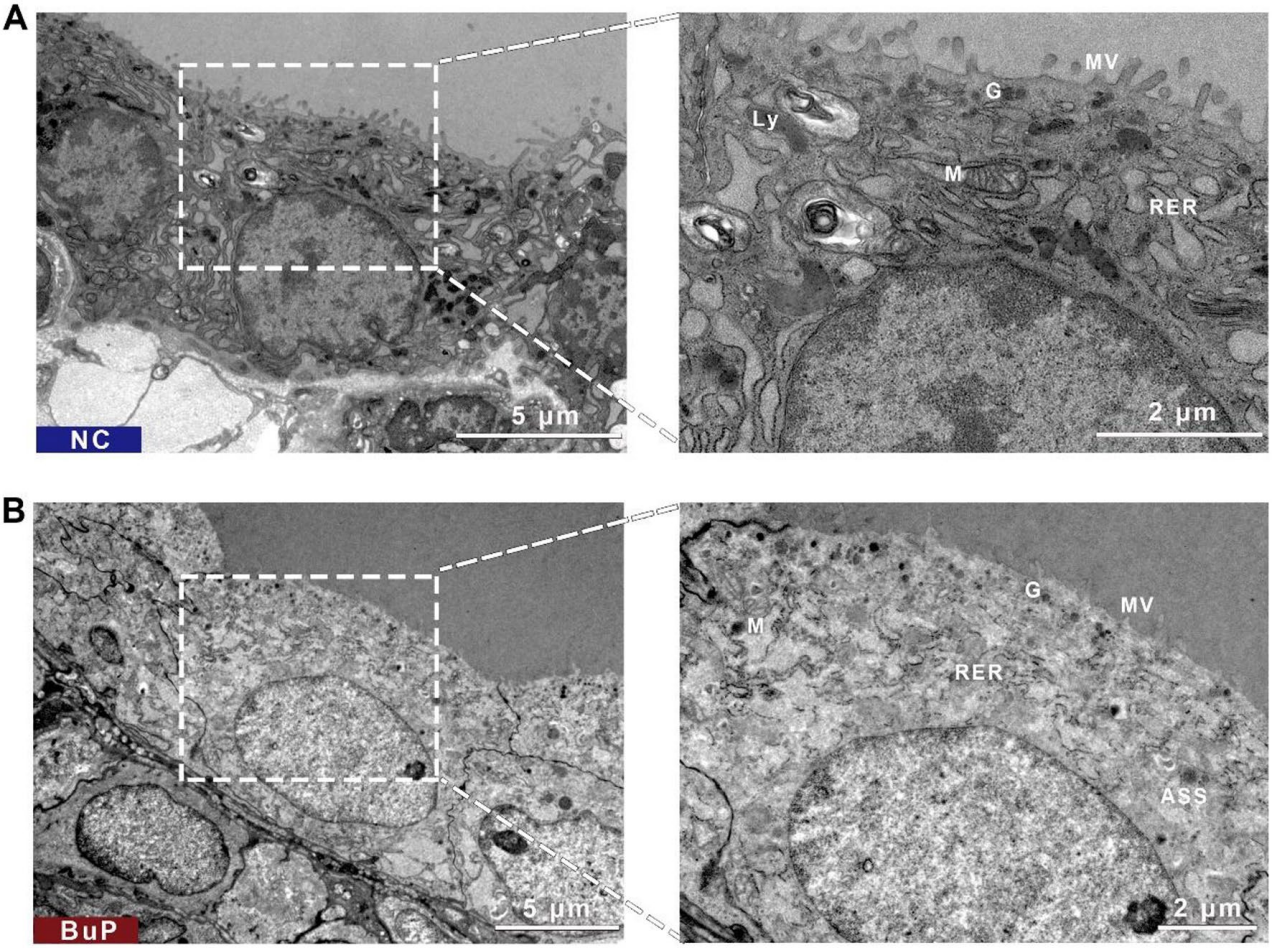
BuP exposure induced ultrastructural changes in thyroid epithelial cells. (**A**) Ultrastructure of normal thyroid epithelial cells from the control group. The mitochondria, rough endoplasmic reticulum, secretory granules, lysosomes, and microvilli are all clear. (**B**) Ultrastructure of thyroid epithelial cells from BuP-exposed rats. The cells were edematous and the organelles were swollen. Mitochondria were enlarged and vacuolated. Rough endoplasmic reticulum was enlarged. Secretory granules were reduced. Microvilli were reduced. Some autophagolysosomes were visible. Transmission electron microscopy. scale bar = 5 µm (left images), 2 µm (right images). n = 5. ***Abbreviations:*** M, mitochondria; RER, rough endoplasmic reticulum; G, secretory granules; Ly, lysosomes; Mv, microvilli; TPO, thyroid peroxidase; Tg, thyroglobulin; ASS , autophagolysosomes.

By electron microscopy, the epithelium of the BuP-exposed rat was ultrastructurally disrupted compared with the epithelium of the control rat (Fig. 3A). BuP-exposed follicular cells were generally columnar and edematous. The intercellular space was narrow. Microvilli were atrophied and shed, resulting in a reduction in the number of microvilli. Organelles were also swollen. Shapes of nuclei were irregular. There were cavities in the local nuclear membrane and heterochromatin was increased in the nucleus. Mitochondria were highly edematous and vacuolated, together with mitochondrial membrane structure disappeared and the number of mitochondrial cristae was reduced. The rough endoplasmic reticulum was enlarged, and many flocculations were seen in the endoplasmic reticulum pool, along with endoplasmic reticulum ribosomes that had fallen off. Many lysosomes, secretory granules and a small number of autophagolysosomes were distributed in the cytoplasm (Fig. 3B).

### BuP disrupted thyroid function and caused hypothyroidism in rats

**Figure 4:**
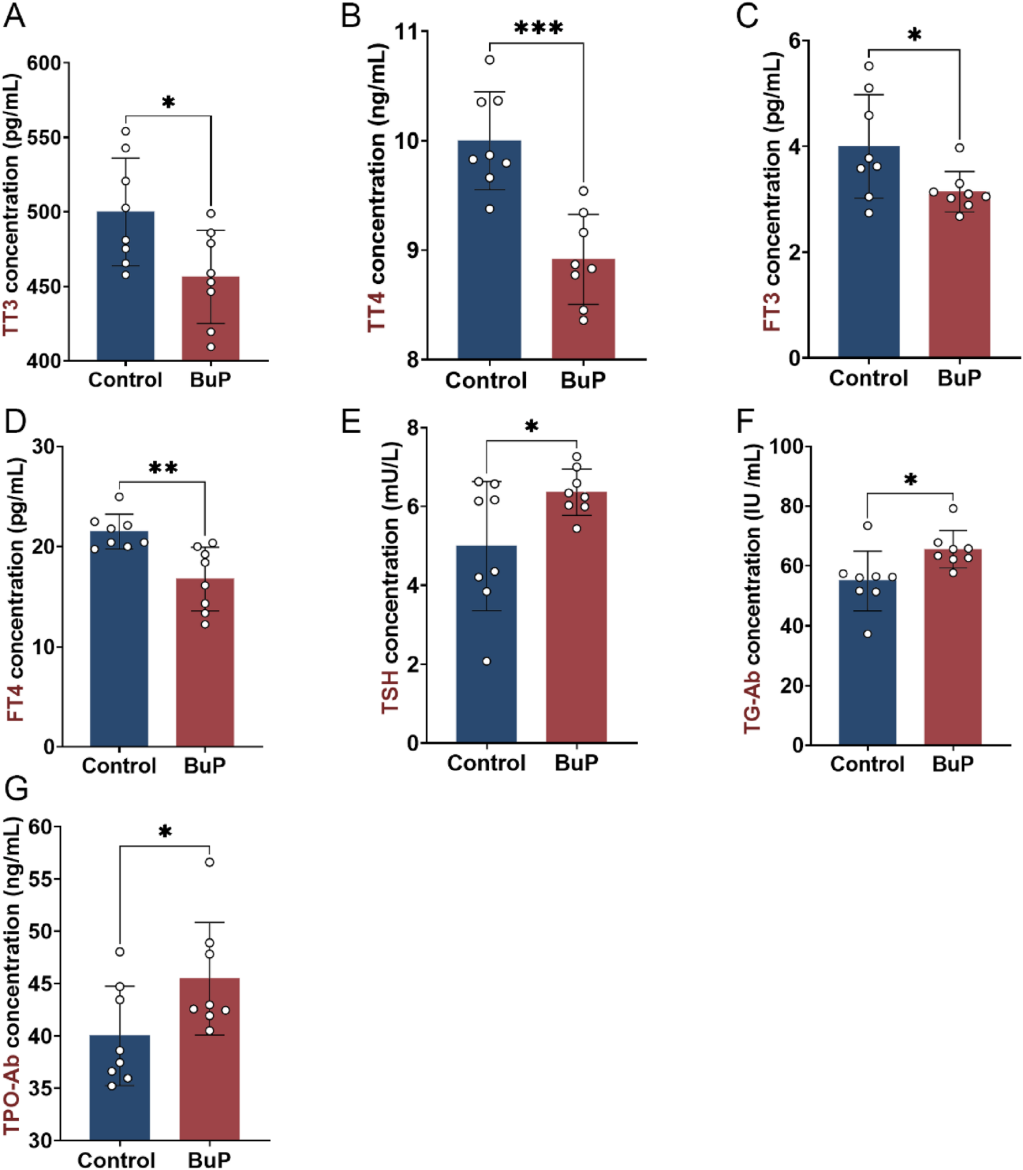
BuP exposure disrupted thyroid hormone levels and caused hypothyroidism in rats. (**A–D**) Serum levels of total triiodothyronine (TT3), total thyroxine (TT4), free T3 (FT3), and free T4 (FT4) were all decreased in the BuP-exposed rats compared with the control rats. (**E**) Serum thyroid-stimulating hormone (TSH) levels were increased in BuP-exposed rats compared with control rats. (**F**, **G**) The levels of autoimmune thyroid antibodies, including thyroglobulin antibody (Tg-Ab) and thyroid peroxidase antibody (TPO-Ab), were increased in the BuP-exposed rats compared with the control rats. n = 8, data are expressed as mean ± SD. Compared with control group, * *P* < 0.05, **P < 0.05, ***P < 0.001.

Serum thyroid hormones, TSH, and autoimmune thyroid antibodies were measured by ELISA. Compared with the control rats, the levels of serum TT3, TT4, FT3, and FT4 were decreased in the BuP-exposed rats (Fig. 4A–D). Correspondingly, the level of TSH was increased (Fig. 4E). Meanwhile, the levels of TPO-Ab and Tg-Ab were also increased in the BuP-exposed rats (Fig. 4F, G).

### Sodium/iodide symporter (NIS) levels were inhibited in BuP-exposed rats

**Figure 5:**
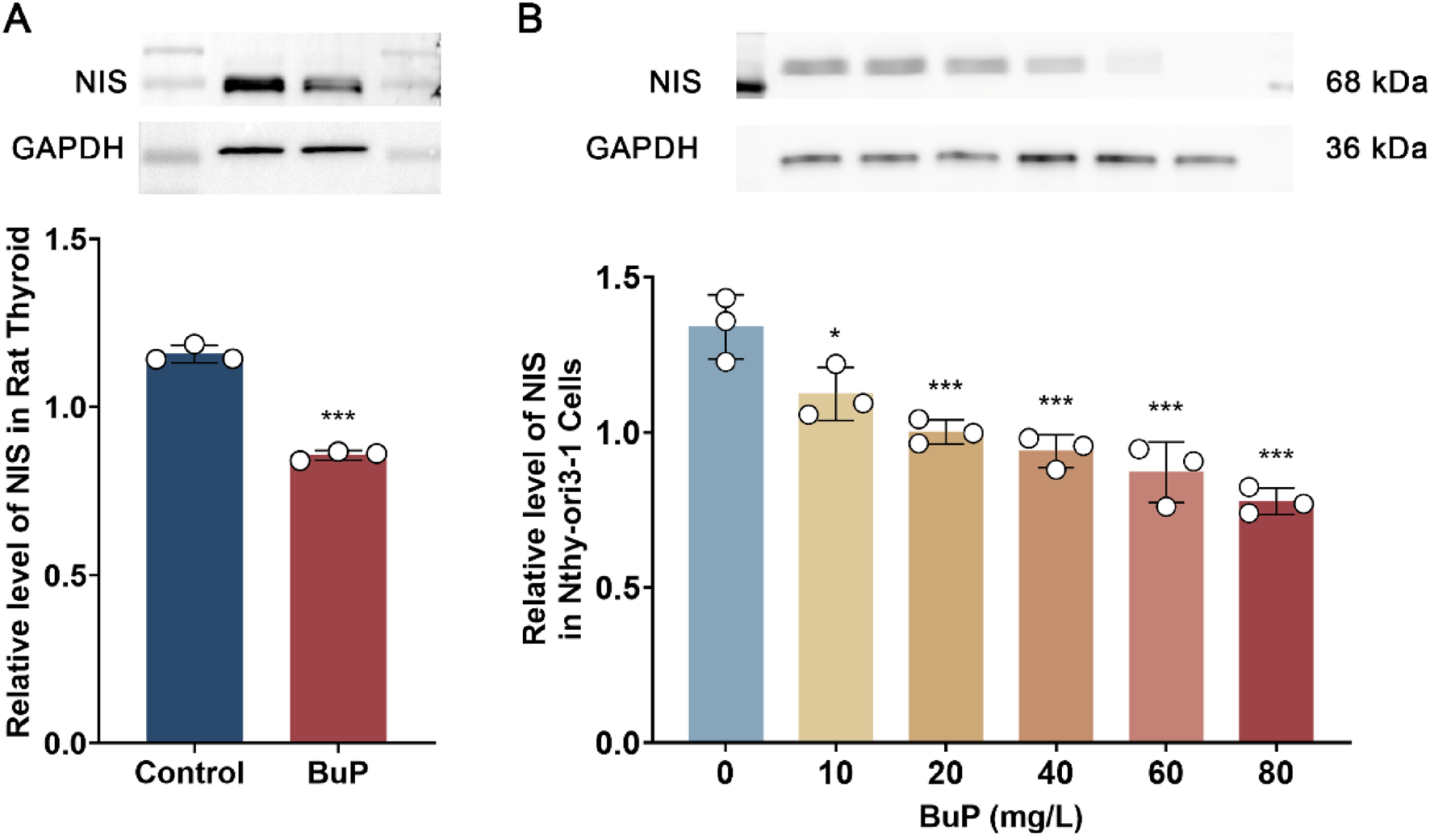
BuP inhibited the level of sodium/iodide symporter (NIS) in rat thyroid and human thyroid cell lines. (**A**) In vivo, BuP exposure caused a decrease in NIS in rats compared to the control group. (**B**) In vitro, BuP inhibited the level of NIS in Nyth-ori3-1, a human thyroid epithelial cell line, in a concentration-dependent manner. n = 3, data are expressed as mean ± SD. Compared with control group, * *P* < 0.05, ***P < 0.001.

Iodide anion (I-) is essential for thyroid hormone synthesis. NIS, also known as the sodium/iodide cotransporter, is located on the membrane of thyroid epithelial cells and is necessary for the transport of I- from the blood circulation into the epithelial cells. In vivo, the level of NIS from the thyroid gland of BuP-exposed rats is lower than that from the thyroid gland of control rats (Fig. 5A). In vitro, BuP inhibited the level of NIS in the human thyroid cell line in a concentration-dependent manner (Fig. 5B).

### BuP impaired cell viability and proliferation of thyroid epithelial cells and induced apoptosis

**Figure 6:**
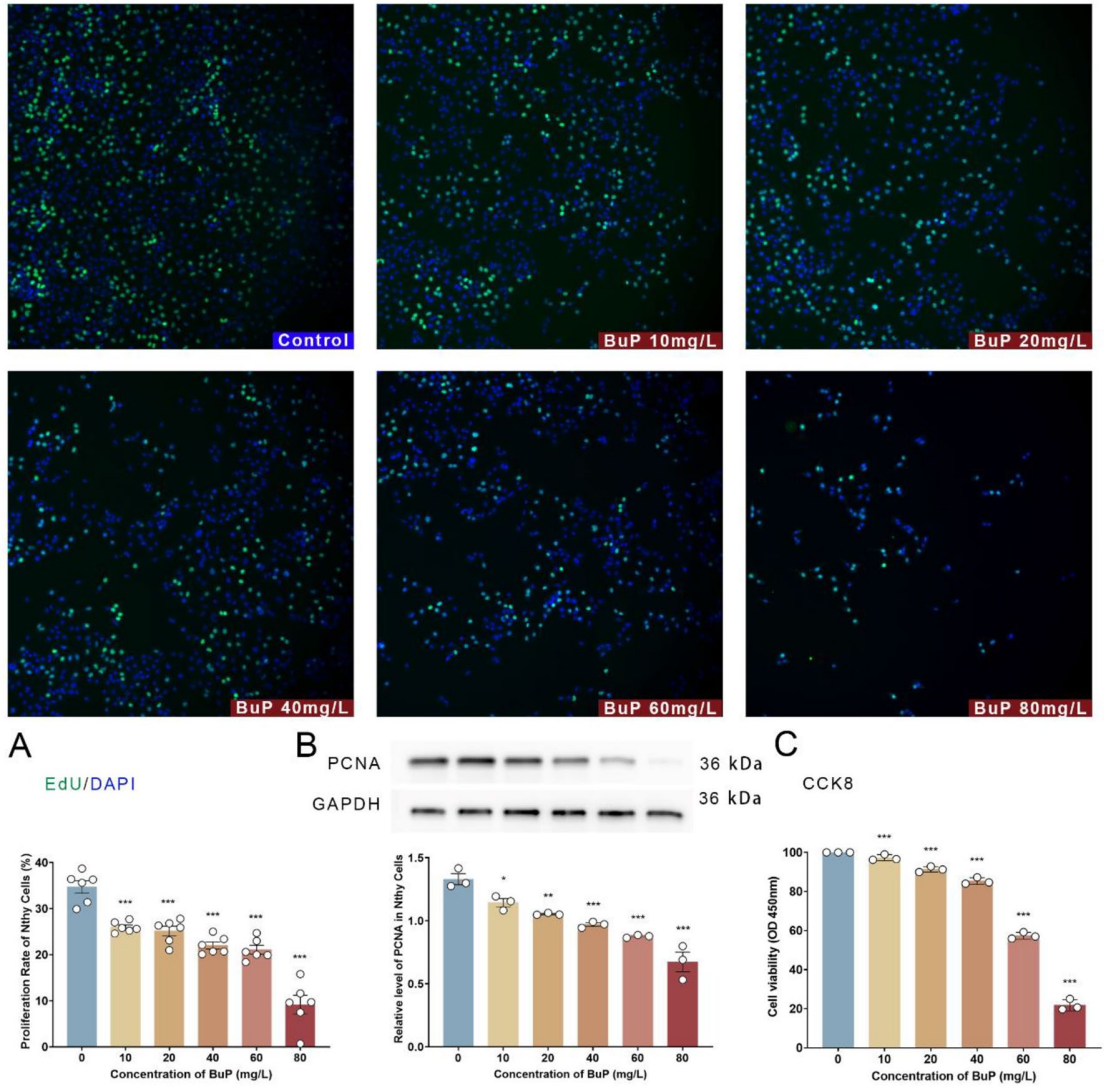
BuP inhibited the proliferation and viability of Nthy cells. (**A**) EdU incorporation assay showed that BuP suppressed the proliferation of Nthy cells in a concentration-dependent manner. (**B**) BuP reduced the level of proliferating cell nuclear antigen (PCNA) in Nthy cells, and the effect was also dose-dependent. (**C**) Cell counting kit-8 (CCK-8) assay showed that cell viability decreased continuously with increasing BuP concentration. n = 6, data are expressed as mean ± SD. Compared with control group, * *P* < 0.05, **P < 0.01, ***P < 0.001.

In vitro, Nyth cells were cultured with 0, 10, 20, 40, 60, and 80 mg/L BuP for 24 h. The cell proliferation rate was determined by EdU incorporation assay and PCNA level. The EdU incorporation assay showed that BuP decreased the proliferation rate of Nyth cells in a concentration-dependent manner (Fig. 6A). The level of proliferating cell nuclear antigen (PCNA) was determined by Western blotting, and the result showed that it was inhibited by BuP treatment, also in a concentration-dependent manner (Fig. 6B). Cell viability was determined by cell counting kit-8 (CCK-8), and the result showed that BuP inhibited it, also in a concentration-dependent manner (Fig. 6C).

**Figure 7:**
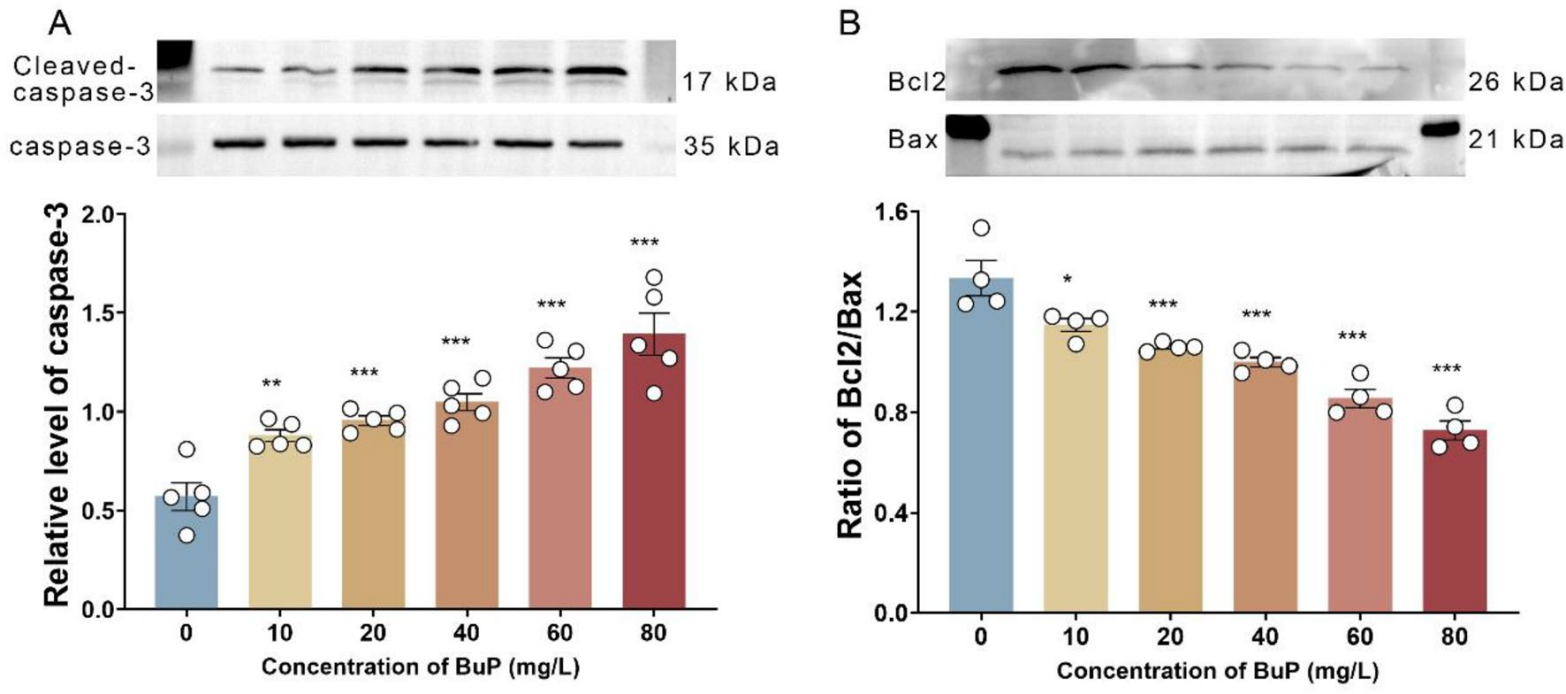
BuP induced apoptosis and impaired the anti-apoptosis ability of thyroid epithelial cells. **(A**) BuP increased the level of cleaved caspase-3, indicating that it promoted apoptosis of thyroid follicular cells. n = 5. (**B**) BuP decreased the level of Bcl-2 and increased the level of Bax, resulting in a decrease in the ratio of Bcl-2/Bax, indicating that BuP decreased the anti-apoptotic ability of thyroid follicular cells. n = 4, data are expressed as mean ± SD. Compared with control group, * *P* < 0.05, **P < 0.01, ***P < 0.001.

Cell apoptosis was determined by the level of cleaved caspase-3 and the ratio of Bcl-2 to Bax. Western blotting showed that the level of cleaved caspase-3 (the active state of caspase-3) in Nyth cells increased with increasing BuP concentration (Fig. 7A), indicating an increase in apoptosis. In contrast, the ratio of Bcl-2 to Bax (Bcl-2/Bax) decreased with increasing concentration of BuP in the culture medium increased (Fig. 7B), indicating that the anti-apoptotic ability of Nyth cells decreased.

## Discussion

As mentioned in the Introduction, PBs have some toxicity to organs, such as the breast, adrenal glands, and gonads. PBs also affect thyroid development in zebrafish, thyroid weight in rodents, and thyroid function in humans. Exposure to BuP increases thyroid peroxidase activity, serum TSH levels, and decreases T4 levels and type 1 iodothyronine deiodinase (D1) activity in rats^25^. When exposed to a higher dose (50 mg/kg bw/day) of BuP for a longer period of time (60 days), rats develop significant hypothyroidism^26^. In vivo concentrations of PBs are associated with abnormal thyroid hormone levels and may also affect the health of offspring through the mother^27^. For example, BuP and PrP can result in decreased TT3 and FT4 and abnormal TSH^28–31^. Maternal exposure to MeP, EtP, and BuP has been associated with maternal thyroid dysfunction, neonatal TSH levels, birth weight and length, and the development of attention deficit hyperactivity disorder (ADHD) in children^30–33^. However, these findings are mainly based on observations of phenomena, and it is still difficult to fully elucidate the mechanism of the effects of PBs on the thyroid.

This study found that high-dose and long-term use of BuP caused abnormalities in thyroid structure and function. BuP exposure resulted in increased thyroid volume and weight in rats. Under light microscopy, the morphology of the thyroid follicles was abnormal and the follicular epithelial cells were swollen. Under electron microscopy, the microvilli at the top of the follicular cell were absent and diminished. The rough endoplasmic reticulum was dilated, ribosomes were lost, and the nuclei were irregular in shape and partially hollow. The serum levels of TT3, TT4, FT3, and FT4 in the rats decreased, and the levels of TSH and thyroid autoantibodies including TG-Ab and TPO-Ab increased. These changes were manifestations of hypothyroidism. In vitro, BuP decreased the sodium/iodide symporter (NIS) level of thyroid epithelial cells, inhibited proliferation and cell viability, and induced apoptosis.

NIS is a symporter that imports ionized iodine into thyroid follicular cells. After entering the thyroid follicular epithelium, ionized iodine is transported to the junction of the microvilli and the follicular lumen and is activated by peroxidase. Ribosomes on the rough endoplasmic reticulum are the site of thyroglobulin (Tg) synthesis, and damage to the ribosome will affect the iodination of Tg tyrosine residues and the synthesis of T3 and T4. In this study, BuP toxicity causes damage to thyroid follicular cells, which may be the cause of morphological changes in thyroid follicular epithelial cells, such as reduction and shedding of microvilli, swelling of rough endoplasmic reticulum, and abnormal nuclear morphology. These organelle morphological changes are consistent with the characteristics of early cell apoptosis. Apoptosis/death of thyroid follicular cells may result in decreased levels of thyroxine, including TT3, TT4, FT3, and FT4, and increased levels of thyrotropin. The mechanism by which BuP causes low thyroxine levels is not fully understood. Hypothyroidism may be caused by interference with the absorption of iodine, biosynthesis, degradation, and metabolism of thyroxine. Further detection and analysis of these biological processes are needed. Apoptosis/death of thyroid follicular epithelial cells also causes autoantigens such as TG and TPO to be released from the cells into the blood, and then the body produces corresponding antibodies against them.

This study has several limitations due to our experimental conditions. First, this study only examined the structural and functional damage to the thyroid gland caused by high-dose exposure to BuP, and there is a lack of data on the effects of different concentrations, especially low doses of BuP, on the thyroid gland. Second, due to the limited experimental conditions, the distribution and concentration of BuP in tissues were not determined in this study. Third, it was not investigated whether BuP directly affects the hypothalamic-pituitary-thyroid axis in the rat. These aspects will be the direction of our future work. Finally, the expressions of thyroid-related genes were not measured in the in vitro experiments (Supplementary Information). Despite the above limitations, this study demonstrates the effects of high-concentration exposure to BuP on mammalian thyroid structure and function and has implications for improving community and individual awareness of environmental paraben exposure.

## Conclusion

This study shows that high-dose exposure to BuP damages the ultrastructure of thyroid follicular cells, thereby affecting thyroid follicles and leading to thyroid dysfunction. Exposure to BuP may be one of the risk factors for hypothyroidism.

### Supplementary Information

Supplementary Figures.

## Data Availability

Data are available to qualified researchers upon reasonable request to the corresponding author, Jun Jiang, at jiangjun@swmu.edu.cn.

## References

[1] Husoy T, Andreassen M, Hjertholm H, Carlsen MH, Norberg N, Sprong C (2019). The Norwegian biomonitoring study from the EU project EuroMix: Levels of phenols and phthalates in 24-h urine samples and exposure sources from food and personal care products. Environ. Int..

[2] Cheng L, Huang K, Cui H, Wang X, Zhang H, Zeng L (2020). Coiled molecularly imprinted polymer layer open-tubular capillary tube for detection of parabens in personal care and cosmetic products. Sci. Total Environ..

[3] Soni MG, Carabin IG, Burdock GA (2005). Safety assessment of esters of p-hydroxybenzoic acid (parabens). Food Chem. Toxicol..

[4] Chung WH, Lin JS, Ding WH (2019). Dual-vortex-assisted matrix solid-phase dispersion coupled with isotope-dilution ultrahigh-performance liquid chromatography-high resolution mass spectrometry for the rapid determination of parabens in indoor dust samples. J. Chromatogr. A..

[5] Feng J, Zhao J, Xi N, Guo W, Sun J (2019). Parabens and their metabolite in surface water and sediment from the Yellow River and the Huai River in Henan Province: Spatial distribution, seasonal variation and risk assessment. Ecotoxicol. Environ. Saf..

[6] Wang N, Hu X, Lu S, Ma S, Kang L, Liao S (2019). Interrelationship of anthropogenic activity and parabens in fish from Taihu Lake during 2009–2017. Environ. Pollut..

[7] Xue J, Kannan K (2016). Accumulation profiles of parabens and their metabolites in fish, black bear, and birds, including bald eagles and albatrosses. Environ. Int..

[8] Leppert B, Strunz S, Seiwert B, Schlittenbauer L, Schlichting R, Pfeiffer C (2020). Maternal paraben exposure triggers childhood overweight development. Nat. Commun..

[9] Jewell C, Prusakiewicz JJ, Ackermann C, Payne NA, Fate G, Voorman R (2007). Hydrolysis of a series of parabens by skin microsomes and cytosol from human and minipigs and in whole skin in short-term culture. Toxicol. Appl. Pharmacol..

[10] Aubert N, Ameller T, Legrand JJ (2012). Systemic exposure to parabens: pharmacokinetics, tissue distribution, excretion balance and plasma metabolites of [14C]-methyl-, propyl- and butylparaben in rats after oral, topical or subcutaneous administration. Food Chem. Toxicol..

[11] Fransway AF, Fransway PJ, Belsito DV, Yiannias JA (2019). Paraben toxicology. Dermatitis..

[12] Moos RK, Apel P, Schroter-Kermani C, Kolossa-Gehring M, Bruning T, Koch HM (2017). Daily intake and hazard index of parabens based upon 24 h urine samples of the German Environmental Specimen Bank from 1995 to 2012. J. Expo. Sci. Environ. Epidemiol..

[13] Boberg J, Taxvig C, Christiansen S, Hass U (2010). Possible endocrine disrupting effects of parabens and their metabolites. Reprod. Toxicol..

[14] Nowak K, Ratajczak-Wrona W, Gorska M, Jablonska E (2018). Parabens and their effects on the endocrine system. Mol. Cell Endocrinol..

[15] Boberg J, Johansson HKL, Axelstad M, Olsen GPM, Johansen M, Holmboe SA (2020). Using assessment criteria for pesticides to evaluate the endocrine disrupting potential of non-pesticide chemicals: Case butylparaben. Environ. Int..

[16] Maske P, Dighe V, Mote C, Vanage G (2020). n-Butylparaben exposure through gestation and lactation impairs spermatogenesis and steroidogenesis causing reduced fertility in the F1 generation male rats. Environ. Pollut..

[17] Kolatorova L, Vitku J, Hampl R, Adamcova K, Skodova T, Simkova M (2018). Exposure to bisphenols and parabens during pregnancy and relations to steroid changes. Environ. Res..

[18] Zhang L, Ding S, Qiao P, Dong L, Yu M, Wang C (2016). n-butylparaben induces male reproductive disorders via regulation of estradiol and estrogen receptors. J. Appl. Toxicol..

[19] Guerra MT, Sanabria M, Cagliarani SV, Leite GA, Borges CD, De Grava KW (2017). Long-term effects of in utero and lactational exposure to butyl paraben in female rats. Environ. Toxicol..

[20] Liang J, Yang X, Liu QS, Sun Z, Ren Z, Wang X (2022). Assessment of thyroid endocrine disruption effects of parabens using in vivo, in vitro, and in silico approaches. Environ. Sci. Technol..

[21] Vo TT, Yoo YM, Choi KC, Jeung EB (2010). Potential estrogenic effect(s) of parabens at the prepubertal stage of a postnatal female rat model. Reprod. Toxicol..

[22] Wu NX, Deng LJ, Xiong F, Xie JY, Li XJ, Zeng Q (2022). Risk of thyroid cancer and benign nodules associated with exposure to parabens among Chinese adults in Wuhan, China. Environ. Sci. Pollut. Res. Int..

[23] Koeppe ES, Ferguson KK, Colacino JA, Meeker JD (2013). Relationship between urinary triclosan and paraben concentrations and serum thyroid measures in NHANES 2007–2008. Sci. Total Environ..

[24] Aker AM, Johns L, McElrath TF, Cantonwine DE, Mukherjee B, Meeker JD (2018). Associations between maternal phenol and paraben urinary biomarkers and maternal hormones during pregnancy: A repeated measures study. Environ. Int..

[25] Gogoi P, Kalita JC (2020). Effects of butylparaben exposure on thyroid peroxidase (TPO) and type 1 iodothyronine deiodinase (D1) in female Wistar rats. Toxicology..

[26] Taha M, Marie AM, Ahmed-Farid OA (2020). Combined approaches for evaluation of xenoestrogen neural toxicity and thyroid dysfunction: Screening of oxido-nitrosative markers, DNA fragmentation, and biogenic amine degradation. J. Biochem. Mol. Toxicol..

[27] Salazar P, Villaseca P, Cisternas P, Inestrosa NC (2021). Neurodevelopmental impact of the offspring by thyroid hormone system-disrupting environmental chemicals during pregnancy. Environ. Res..

[28] Aker AM, Watkins DJ, Johns LE, Ferguson KK, Soldin OP, Anzalota Del Toro LV (2016). Phenols and parabens in relation to reproductive and thyroid hormones in pregnant women. Environ. Res..

[29] Berger K, Gunier RB, Chevrier J, Calafat AM, Ye X, Eskenazi B (2018). Associations of maternal exposure to triclosan, parabens, and other phenols with prenatal maternal and neonatal thyroid hormone levels. Environ. Res..

[30] Hu L, Mei H, Cai X, Hu X, Duan Z, Liu J (2023). Maternal paraben exposure and intra-pair thyroid-stimulating hormone difference in twin neonates. Ecotoxicol. Environ. Saf..

[31] Kang S, Shin B, Kwon J, Park E, Kim BJEE (2019). Effects of prenatal exposure to triclosan and parabens on thyroid hormones levels during pregnancy and birth outcomes: from the Mother and Kids Environmental health (MAKE) Study. Environ. Epidemiol..

[32] Baker BH, Wu H, Laue HE, Boivin A, Gillet V, Langlois MF (2020). Methylparaben in meconium and risk of maternal thyroid dysfunction, adverse birth outcomes, and Attention-Deficit Hyperactivity Disorder (ADHD). Environ. Int..

[33] Li W, Guo J, Wu C, Zhang J, Zhang L, Lv S (2020). Effects of prenatal exposure to five parabens on neonatal thyroid function and birth weight: Evidence from SMBCS study. Environ. Res..

