# Supplementary figures and images for: High-dose exposure to butylparaben impairs thyroid ultrastructure and function in rats
Source: Sci Rep. 2024 Feb 24;14:4550. doi: 10.1038/s41598-024-55096-4 (PMC10894246; doi:10.1038/s41598-024-55096-4)

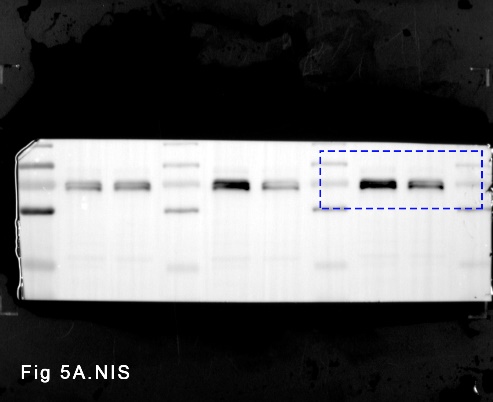

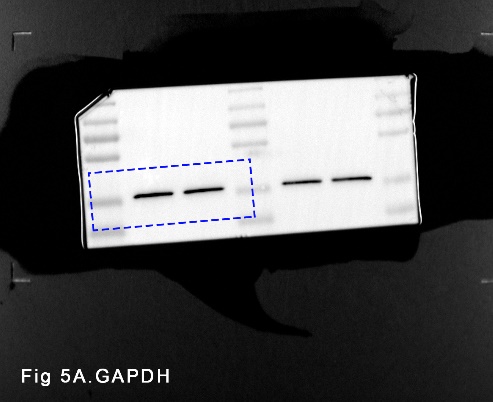


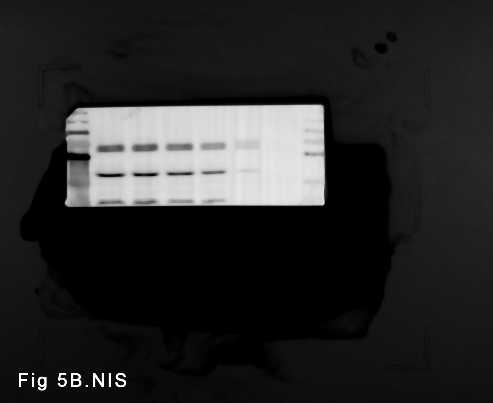

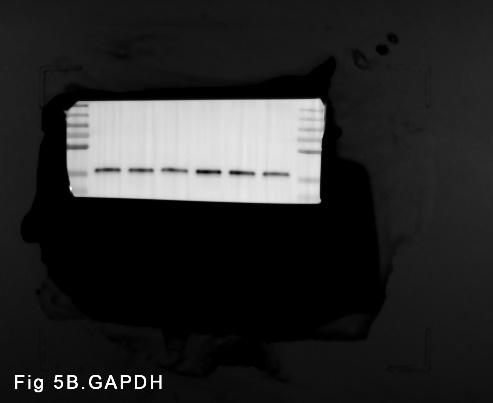


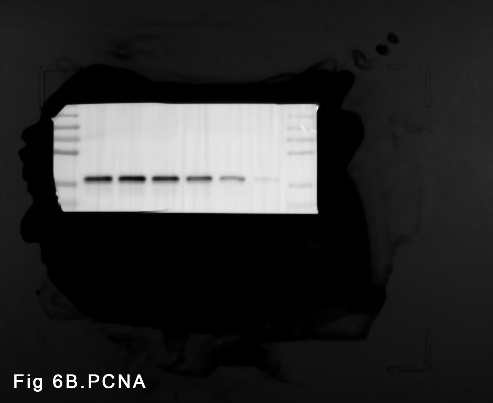

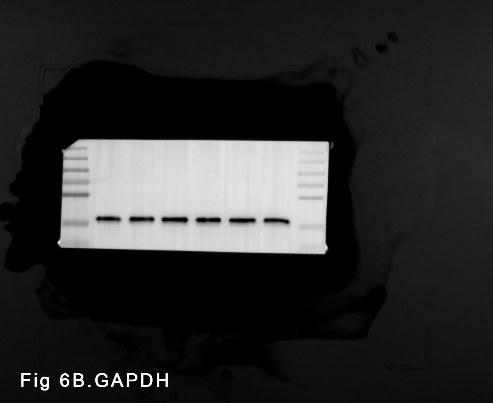


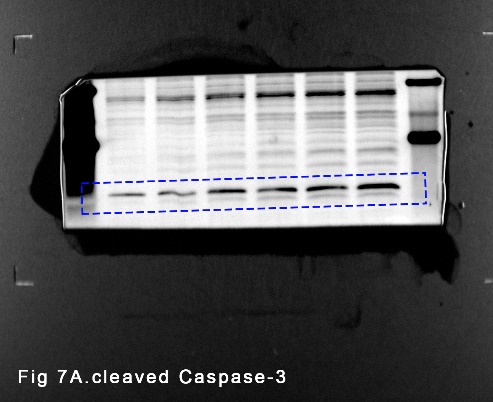

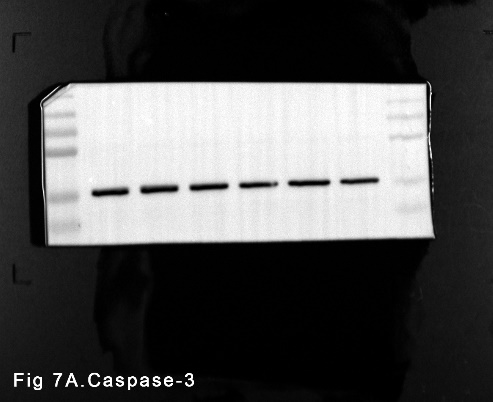


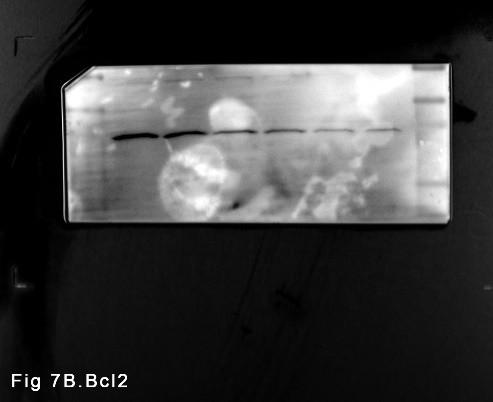

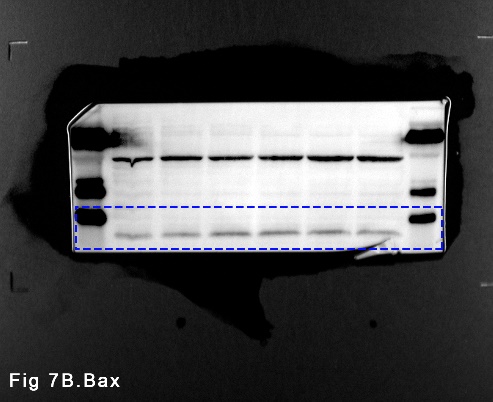

Supplement: Supplementary file 1 — Supplementary Figures. [file 41598_2024_55096_MOESM1_ESM.docx]
